# The effectiveness of intervention with cognitive behavioral therapy on pornography: A systematic review protocol of randomized clinical trial studies

**DOI:** 10.1002/hsr2.341

**Published:** 2021-08-05

**Authors:** Aida Lotfi, Masoudeh Babakhanin, Masumeh Ghazanfarpour

**Affiliations:** ^1^ Department of Medicine Qazvin University of Medical Sciences Qazvin Iran; ^2^ Social Determinants of Health Research Center Semnan University of Medical Sciences and Health Services Semnan Iran; ^3^ School of Nursing and Midwifery Mashhad University of Medical Sciences Mashhad Iran

**Keywords:** cognitive behavioral therapy, compulsive sexual behavior, problematic pornography use, protocol

## Abstract

**Background:**

The increasing rate of problematic pornography use (PPU) among the general population has risen. There are limited data on the efficacy of Cognitive Behavioral Therapy (CBT) on online pornographic addiction; therefore, this study aimed at investigating the issue.

**Methods:**

SCOPUS, PubMed, PubPsych, WOS (Web of Science), Cochrane Central Register of Controlled Trials, Google Scholar, Scientific Information Database (SID) & Iranmedex, and other databases (for gray literature) (eg, conference papers, key journals) will be systematically reviewed. Preliminary search strategies were started on March 2, 2019, and will be updated in April 2021. Eligibility criteria were having PPU, with designs of interest including randomized trials with three or more months of follow‐up with CBT intervention. Two authors will independently conduct data extraction and quality assessment. A modified Verhagen checklist for clinical trial studies will be used for quality assessment. Pooled measures of association will be computed using random‐effects model meta‐analyses. Between‐study heterogeneity will be assessed using the I2 statistic and the Cochrane χ2 statistic. Minor study effects will be evaluated for meta‐analyses with sufficient studies using funnel plots and Egger's test. If a meta‐analysis is appropriate, quantitative data will be pooled using the comprehensive meta‐analysis software.

**Discussion:**

The evidence obtained in this meta‐analysis will help to determine whether CBT can decrease PPU severity, anxiety, depression, and compulsive sexual behavior. In addition, due to the comprehensive view on CBT effects on PPU patients, which was not clarified before, we can expect that the results of this study will benefit psychiatrists.

**List of Abbreviations**
CBT: cognitive‐behavioral therapy
CI: confidence Interval
CSB: compulsive sexual behavior
GRADE: grades of recommendation assessment, development, and evaluation
PICOS: population/intervention/comparison/outcomes/study design
PPU: problematic pornographic use
PRISMA: preferred reporting items for systematic reviews and meta‐analyses
PRISMA‐P: preferred reporting items of systematic reviews and meta‐analysis for protocols
RCT: randomized controlled trial
SMDs: standardized mean differences

## 1 BACKGROUND

The increasing rate of pornography use among the general population has caused mental health professionals to be deeply concerned about the increased number of patients presenting the symptoms of sexual addictions and sexual compulsivity. Studies have revealed that watching pornography is related to many negative consequences, such as failure in the relationship and sexual satisfaction, impairment of academic and professional functioning, subjective distress, perceived addiction, and sexual compulsivity.^1^, ^2^, ^3^ Due to accessibility to the Internet worldwide and feasible access to pornographic websites, the number of people affected by online pornography is rapidly increasing. In this case, it is reported that the number of people using adult pornographic videos has been doubled from 1992 to 2006.^4^ In addition, the youth are the most sensitive group of society in this rapidly increasing wave of pornography use. The majority of college students (males and females) watched pornographic videos at least one time in their lifetime (approximately 90%), and almost half of them (40%) reported watching pornography weekly.^5^ As a result, the increasing prevalence of online pornography use among adolescents (mostly male) revived social worries regarding the possible effects of pornography use on sexual aggressiveness.^6^ The growing rate of psychological addiction and compulsive pornography use as a prevalent complication could be accompanied by considerable psychological distress forms, such as depression, anxiety, perceived stress, and anger.^7^

Based on the DSM‐5 criteria classification, problematic pornographic use (PPU) is characterized by excessive sexual behaviors, diminished self‐control over sexual engagement, use of sex for escaping from or avoiding negative emotions, and functional impairment and distress.^8^ Furthermore, other features that are not included in DSM‐5 and are related to addictive pornography use are (a) frequent, excessive, or compulsive behavioral engagement; (b) an appetitive urge before engaging in the behavior to reach/maintain a positive emotional state or to escape from/avoid a negative emotional state; (c) diminished self‐control over behavioral engagement; and (d) continued attention despite adverse consequences, which, in turn, leads to significant personal distress and functional impairment.^9^

The prevalence of PPU in 2019 was reported 1% to 6% in the adult population.^10^ However, there is no particular treatment for this issue, and treatment strategies targeting sexual addiction are vast. They could be included in medications, behavior therapies, 12‐step approaches, and solution‐focused therapy.^11^ Cognitive‐behavioral therapy (CBT) is typical and might be the most effective technique for such patients addicted to pornography. The CBT has demonstrated generally good tolerability, acceptability, and efficacy for other conditions like migraines in children.^12^

Furthermore, CBT as a comprehensive treatment and an isolated method was reported to be effective for compulsive sexual behaviors. Therefore, it might not be surprising that CBT is the chosen intervention for 81% of addiction counselors.^13^ The CBT method mainly has focused on maladaptive thinking patterns and the associated beliefs and behaviors supporting those patterns.^14^ CBT addresses distorted thinking, and by using disregarded or ignored empirical evidence in the patient's life, it reframes those thoughts. The reframing becomes an affirmation for patients challenging those aspects of a belief system that might be irrational.^15^

Previous reports on the efficacy of this method on sex‐addicted patients combined with motivational interviewing, several open studies and case reports have reported significant reductions in sexual behaviors, such as frequency of sexual partners and amount of time spent online during working hours.^16^, ^17^, ^18^ The isolated use of the CBT method in an open study revealed that by using this method, the overall depression, anxiety, and sexually compulsive behaviors of patients were reduced.^18^ Recently, a literature review evaluating the effects of psychological methods on PPU has revealed that the use of CBT improved quality of life and reduced depressive symptoms; in addition, most clients managed their presenting complaints.^19^

A randomized clinical trial (RCT) framework demonstrated positive and long‐term effects on PPU in the intervention group in a survey of the CBT‐based method.^3^

Primary outcome: Time spent on watching pornography.

Secondary outcome: Psychological symptoms (eg, mood and Obsessive‐compulsive disorder symptoms, including obsessions, compulsions, or both) for watching pornography.

### 1.1 Research aims

This study aims at investigating the impact of cognitive‐behavioral therapy on pornography.

## 2 METHODS AND ANALYSIS

The preliminary search strategy will be based on Population/Intervention/Comparison/Outcomes/Study Design (PICOS), PRISMA‐P checklist,^19^, ^20^ and the acronym with this review question: Does Cognitive Behavioral Therapy intervention increase/decrease related symptoms in a group with PPU?

The PROSPERO—International Prospective Register of Systematic Reviews—Registration Number (CRD) for this study is 42020161679 and available at https://www.crd.york.ac.uk/prospero/display_record.php?ID=CRD42020161679.

The six databases, including SCOPUS, PubMed, PubPsych, WOS (Web of Science), Cochrane Central Register of Controlled Trials, Google Scholar, Scientific Information Database (SID) & Iranmedex, and other databases (for gray literature) (eg, conference papers, key journals), will be systematically reviewed from the onset of databases searching until the date of the current search. Furthermore, there will be no restrictions regarding the language. The preliminary search strategy was implemented on March 2, 2019 and will be updated in March 2021.

The search strategy will be consisted of critical terms according to a pre‐established PICOS acronym. Two researchers (M.B. and A.L.) will independently implement the search strategy in all databases. The bibliographic software EndNote 8.00 (https://www.myendnoteweb.com/) will be used to store all the references and comprehensive searches. It should be noted that all keywords in each database (such as MeSH terms, PsycINFO Thesaurus, and mesh browser) will be identified with their synonyms. The search terms were combined using the Boolean operators “AND” and “OR.”^21^ (Table 1).

**TABLE 1:** Concepts and search items

Databases	Search items
Pubmed	((Pornography [Tiab]) OR Eroticism [Tiab]) OR “Internet pornography use” [tiab] OR “online pornography” [Tiab] OR “Internet sex addiction” OR “Internet pornography” [tiab] OR cyberpornography [Tiab] AND (cognitive behavioural therapy [Tiab]) OR (CBT [Tiab])
SCOPUS	([TITLE‐ABS]Pornography) OR TITLE‐ABS (Eroticism) OR TITLE‐ABS (“Internet pornography use”) OR TITLE‐ABS (“Internet pornography”) OR TITLE‐ABS (cyberpornography) AND TITLE‐ABS (cognitive AND behavioral therapy) OR (CBT)
WOS	TS = Pornography OR TS = (Eroticism) OR TS = (Internet pornography use) OR TS = (Internet pornography) OR TS = (cyberpornography) AND TS = (cognitive AND behavioral therapy) OR TS = (CBT)
PsycINFO	“Pornography” (all fields) OR “Eroticism” (all fields) OR “Internet pornography use” “cyberpornography” OR (MeSH terms) AND “cognitive AND behavioral therapy” (all fields) (MeSH terms)

Study selection will be based on the PICOS^22^ search strategy shown in Table 2. Only RCT studies investigated the effect of CBT on pornography use in both sexes; all ages and ethnicities will be included in the study. Observational studies, N RCT, and qualitative studies will be excluded.

**TABLE 2:** Inclusion and exclusion criteria

PICOS acronym	Inclusion criteria	Exclusion criteria
P—Population	Both sexes, all ages and any ethnicity	None
I—Intervention/Exposure	CBT	Other psychological interventions
C—Comparison	Pre‐ and post‐comparison with a control group or non‐pornography user group	Case report
O—Outcome	Time spent on pornography watching	Studies that report the prevalence and severity of symptoms related to pornography use and people who had mental problems by other causes than pornography use
S—Study Design	RCT	Observational studies, N RCT, qualitative studies
Language	All languages	None

### 2.1 Screening and data extraction

Two independent researchers (A.L. and M.B.) will perform the screening of titles and abstracts in the study. In this process, if disagreement exists, a third reviewer (M.G.) makes a final decision. The standardized form in the Excel software will be used for data extraction, which included four items: (a) identification of the study (study title, first author, country of the study, year of publication), (b) methodological characteristics (study design, sample size, age, sex, groups, controls, recruitment methods, stated length of follow‐up, validated measures, and quality assessment), (c) main findings, and (d) conclusions. Two reviewers will maintain the data extraction independently.

### 2.2 Methodological appraisal

Critical appraisal for assessing each paper's methodological quality will be performed using a modified Verhagen checklist.^23^ The Verhagen checklist consists of eight items as follows: (a) method of randomization and allocation concealment, (b) similarity of prognostic indicators at baseline, (c) considering eligibility criteria, (d) blinding of assessors, (e) providers (f) participants, (g) considering point estimates for outcome measures, and (h) intention‐to‐treat analysis. The score of each item will be 1 and 0 for “Yes” and “No/do not know” answers, respectively. Papers scored at least 5 (out of 8) will be included in the systematic review.^23^ Two independent reviewers (M.B. and A.L.) will assess the methodological quality of eligible trials. Any discrepancies between raters will be discussed until a consensus is reached.

### 2.3 Data synthesis and meta‐analysis

Two independent reviewers (A.L. and M.B.) will insert the extracted data from each study into an Excel sheet. If a meta‐analysis is appropriate, quantitative data will be pooled using the comprehensive meta‐analysis software STATA version 13.0. Analysis of subgroups or subsets will be conducted if the subgroup analysis (eg, age, disease status, ethnicity, socioeconomic status, presence, or absence of comorbidities) was satisfying.

Standardized mean differences (SMDs) and 95% confidence interval (CI) will be used to calculate the effect sizes using a random‐effects model. Heterogeneity will be tested by the I2 statistic (0%‐40%, 30%‐60%, 50%‐90%, and 75%‐100%) indicate potentially low heterogeneity, moderate heterogeneity, substantial heterogeneity, and considerable heterogeneity, respectively). Cohen's proposal will be recruited to interpret the effect size, which corresponds to 0.20, 0.50, and 0.80, considering the small, medium, and high sizes.^24^

### 2.4 Assessment of publication bias

Publication bias will be assessed using Begg and Egger tests.^25^ In addition, a funnel plot will be performed.

### 2.5 Quality of evidence

We will use the Grades of Recommendation Assessment, Development, and Evaluation (GRADE) methodology to rate the overall quality of evidence.^26^ The evidence level will be downgraded due to a risk of detection bias (risk of bias), inconsistency, indirectness, and imprecision through four categories: high, moderate, low, and very low.

### 2.6 Patient and public involvement

No patients or the public will be involved.

### 2.7 Ethics and dissemination

Findings will be disseminated through peer‐reviewed publications.

## 3 DISCUSSION

This study will assess the effectiveness of CBT in PPU patients. Qualitative and quantitative evidence will be summarized on this topic, providing an overview of the current body of knowledge on pornography use. A meta‐analysis will be conducted, and its results will be used to compute standard effect sizes and significance. The effect size, robustness, and quality of evidence obtained in this meta‐analysis will help to determine whether CBT can decrease PPU severity, anxiety, depression, and compulsive sexual behavior (CSB). It is expected that the results will contribute to mental health guidelines. Furthermore, due to the comprehensive view on CBT effects on PPU patients, which was not clarified before, we can expect that the results of the present study will benefit psychiatrists as a treatment option. Lack of access to some databases due to subscription restrictions and few RCT studies in PPU is considered the limitations of our study.

## AUTHORS' CONTRIBUTIONS

Conceptualization: Masoudeh Babakhanian.

Investigation: Masoudeh Babakhanin.

Methodology: Masoudeh Babakhanin.

Project Administration: Masoudeh Babakhanin.

Software: Masoudeh Babakhanin.

Supervision: Masoudeh Babakhanin.

Validation: Masoudeh Babakhanin.

Visualization: Aida Lotfi.

Writing – Original Draft: Aida Lotfi, Masumeh Ghazanfarpour; Masoudeh Babakhanin.

Writing – Review & Editing: Aida Lotfi, Masumeh Ghazanfarpour; Masoudeh Babakhanin.

All authors have read and approved the final version of the manuscript.

Masoudeh Babakhanin had full access to all the data in this study and takes complete responsibility for the integrity of the data and the accuracy of the data analysis.

## CONFLICT OF INTEREST

There is no conflict of interest. The work has not been published previously.

## References

[1] Rosenberg H , Kraus S . The relationship of “passionate attachment” for pornography with sexual compulsivity, frequency of use, and craving for pornography. Addict Behav. 2014;39(5):1012‐1017. 24613495 10.1016/j.addbeh.2014.02.010

[2] Grubbs JB , Volk F , Exline JJ , Pargament KI . Internet pornography use: perceived addiction, psychological distress, and the validation of a brief measure. J Sex Marital Ther. 2015;41(1):83‐106. 24341869 10.1080/0092623X.2013.842192

[3] Minarcik J . Proposed Treatment of Problematic Pornography Use: A Cognitive‐Behavioral Approach. 2016.

[4] Ropelato J . 2005 US pornography industry revenue statistics TopTenREVIEWS. 2006. http://internet-filter-review.toptenreviews.com/internet-pornography-statistics.html

[5] O'Reilly S , Knox D , Zusman ME . College student attitudes toward pornography use. Coll Stud J. 2007;41(2):402‐407.

[6] Dawson K , Tafro A , Štulhofer A . Adolescent sexual aggressiveness and pornography use: a longitudinal assessment. Aggress Behav. 2019;45(6):587‐597. 31432547 10.1002/ab.21854

[7] Grubbs JB , Stauner N , Exline JJ , Pargament KI , Lindberg MJ . Perceived addiction to internet pornography and psychological distress: examining relationships concurrently and over time. Psychol Addict Behav. 2015;29(4):1056. 26372200 10.1037/adb0000114

[8] Schreiber LR , Odlaug BL , Grant JE . 13 compulsive sexual behavior: phenomenology and epidemiology. The Oxford Handbook of Impulse Control Disorders. Oxford, England: Oxford University Press; 2011:165.

[9] Potenza MN . Should addictive disorders include non‐substance‐related conditions? Addiction. 2006;101:142‐151. 16930171 10.1111/j.1360-0443.2006.01591.x

[10] Lewczuk K , Wojcik A , Gola M . Increase in the Prevalence of Online Pornography Use–Objective Data Analysis from the Period between 2004 and 2016 in Poland. 2019. 10.1007/s10508-021-02090-w PMC8888374 34750777

[11] Wilson M . A comparative study of art therapy and cognitive behavioral therapy in the treatment of sexually addictive behaviors and an investigation into the relationship between shame and sexually addicted behaviors in adults. Disser Abstr Int: Sci Eng. 2010;71:2706.

[12] Ng QX , Venkatanarayanan N , Kumar L . A systematic review and meta‐analysis of the efficacy of cognitive behavioral therapy for the management of pediatric migraine. Headache. 2017;57(3):349‐362. 10.1111/head.13016 28028812

[13] Eliason MJ , Arndt S , Schut A . Substance abuse counseling: what is treatment as usual. J Addict Dis. 2005;24(Suppl 1):33‐51.15784522

[14] Corey G . Theory and Practice of Counseling and Psychotherapy. Tehran: Nelson Education; 2015.

[15] Beck J . CBT: Basics and beyond. New York, NY: Guilford Press; 2011.

[16] Shepherd L . Cognitive behavior therapy for sexually addictive behavior. Clin Case Stud. 2010;9(1):18‐27.

[17] Orzack MH , Voluse AC , Wolf D , Hennen J . An ongoing study of group treatment for men involved in problematic internet‐enabled sexual behavior. Cyberpsychol Behav. 2006;9(3):348‐360. 16780403 10.1089/cpb.2006.9.348

[18] Del Giudice MJ , Kutinsky J . Applying motivational interviewing to the treatment of sexual compulsivity and addiction. Sex Addict Compuls. 2007;14(4):303‐319.

[19] Sniewski L , Farvid P , Carter P . The assessment and treatment of adult heterosexual men with self‐perceived problematic pornography use: a review. Addict Behav. 2018;77:217‐224. 29069616 10.1016/j.addbeh.2017.10.010

[20] Moher D , Shamseer L , Clarke M , et al. Preferred reporting items for systematic review and meta‐analysis protocols (PRISMA‐P) 2015 statement. Syst Rev. 2015;4(1):1. 25554246 10.1186/2046-4053-4-1 PMC4320440

[21] Topi H , Lucas W . Mix and match: combining terms and operators for successful web searches. Inf Process Manag. 2005;41(4):801‐817.

[22] Methley AM , Campbell S , Chew‐Graham C , McNally R , Cheraghi‐Sohi S . PICO, PICOS and SPIDER: a comparison study of specificity and sensitivity in three search tools for qualitative systematic reviews. BMC Health Serv Res. 2014;14(1):579. 25413154 10.1186/s12913-014-0579-0 PMC4310146

[23] Verhagen AP , De Vet HC , De Bie RA , et al. The Delphi list: a criteria list for quality assessment of randomized clinical trials for conducting systematic reviews developed by Delphi consensus. J Clin Epidemiol. 1998;51(12):1235‐1241. 10086815 10.1016/s0895-4356(98)00131-0

[24] Deeks JJ , Higgins JP , Altman DG , Group CSM . Analysing data and undertaking meta‐analyses. Cochrane Handbook for Systematic Reviews of Interventions. Hoboken, NJ: John Wiley & Sons; 2019:241‐284.

[25] Egger M , Smith GD , Schneider M , Minder C . Bias in meta‐analysis detected by a simple, graphical test. BMJ. 1997;315(7109):629‐634. 9310563 10.1136/bmj.315.7109.629 PMC2127453

[26] Balshem H , Helfand M , Schünemann HJ , et al. GRADE guidelines: 3. Rating the quality of evidence. J Clin Epidemiol. 2011;64(4):401‐406. 21208779 10.1016/j.jclinepi.2010.07.015

